# Prosthetic rehabilitation of oral submucous fibrosis patients: A systematic review of published case reports and case series

**DOI:** 10.1371/journal.pone.0184041

**Published:** 2017-09-06

**Authors:** Shankargouda Patil, Sachin Chakradhar Sarode, Gargi S. Sarode, Shilpa Bhandi, Kamran Habib Awan, Marco Ferrari

**Affiliations:** 1 Department of Medical Biotechnologies, School of Dental Medicine, University of Siena, Italy; 2 Department of Maxillofacial Surgery and Diagnostic Sciences, Division of Oral Pathology, College of Dentistry, Jazan University, Jazan, Saudi Arabia; 3 Department of Oral Pathology and Microbiology, Dr. D.Y. Patil Dental College and Hospital, Dr. D.Y. Patil Vidyapeeth, Sant-Tukaram Nagar, Pimpri, Pune; 4 Department of Restorative Dental Sciences, Division of Operative Dentistry, College of Dentistry, Jazan University, Jazan, Saudi Arabia; 5 College of Dental Medicine, Roseman University of Health Sciences, South Jordan, Utah, United States; 6 Department of Prosthodontics & Dental Materials and Dean, School of Dental Medicine, University of Siena, Italy; University of Washington, UNITED STATES

## Abstract

**Background:**

Oral submucous fibrosis (OSF) is an insidious chronic condition characterized by restricted mouth opening. Prosthetic rehabilitation is challenging for OSF patients as obtaining a good impression requires adequate mouth opening. The aim of the present review is to systematically present the data from case reports published in the English-language literature.

**Method:**

A comprehensive search of the literature databases (PubMed, Medline, SCOPUS, Web of Science and Google Scholar) along with the references of published articles on prosthetic rehabilitation in OSF patients published to date was conducted. Keywords included a combination of ‘Oral submucous fibrosis’, ‘prosthesis’, ‘dentures’ and/or ‘restricted mouth opening’. Citations from selected references and bibliographic linkages taken from similar cases were included in this review. The inclusion criteria selected for case reports on prosthetic rehabilitation in OSF patients, and cases of restricted mouth opening due to causes other than OSF were excluded from the study.

**Results:**

A total of 21 cases were identified and analysed from 17 papers published in the English-language literature. Of these, 9 cases employed the sectional denture technique, 4 cases emphasized the need-based treatment approach in which conventional methods were modified, and 4 cases used mouth exercising devices. Finally, 1 case each involved, flexible denture, oral screen prosthesis, oral stents, surgery in conjunction with dentures.

**Conclusion:**

Prosthetic rehabilitation in OSF patients is a multifaceted approach and should be patient specific, although sectional dentures have achieved the best results.

## Introduction

Oral submucous fibrosis (OSF) is a chronic oral disease caused by betel nut chewing. It is initially associated with vesicle formation in the oral cavity, followed by inflammatory reactions juxta epithelially that cause stiffness of the oral mucosa, leading to trismus and difficulty eating [1]. Limited mouth opening is the chief complaint of most moderate-to-severe OSF patients and is a definite hindrance to the successful application of prosthetic treatments.

Over the years, a number of rehabilitation techniques to counter limited mouth opening in OSF patients have been tested, including surgeries with splints, use of dynamic mouth opening devices, mouth exercising devices (MEDs), or magnetic attachments, and modifications of conventional denture designs [2]. To design a good prosthesis, a detailed impression of the oral tissues and record of the appropriate anatomic landmarks is required; thus, a precise diagnostic impression must be obtained. It is essential to obtain a diagnostic cast for the development of custom trays and the final impressions [3]. A well-fitted prosthesis helps OSF patients with better esthetics, function and overall well-being, which is a rewarding experience for a prosthodontist [2]. This review highlights all the prosthetic rehabilitation procedures carried out in OSF patients to obtain insight into the best treatment modalities available.

## Methods

### Focused question

What are the different prosthetic techniques and prosthetic devices used during the prosthetic rehabilitation of OSF?

### Search strategy and selection criteria

This systematic review was conducted according to the guidelines of the preferred reporting items for systematic reviews and meta-analyses statement. (S1 Table) A comprehensive search of the literature databases (PubMed, Medline, SCOPUS, Web of Science and Google Scholar) along with cross-references to published articles on prosthetic rehabilitation in patients with OSF from inception to March 2017 was made. Keywords included a combination of ‘Oral submucous fibrosis’, ‘prosthesis’, and/or ‘restricted mouth opening’. Citations from selected references and bibliographic linkages taken from similar cases were included in this review. Journals related to subjects such as prosthodontics, oral pathology and oral surgery were also searched using the aforementioned keywords.

### Eligibility criteria

Original English-language case reports and case series on prosthetic rehabilitation in OSF patients were included in the present systematic review. By contrast, cases of limited mouth opening due to causes other than OSF were excluded from the study. The extracted data included date of publication, author name, number of cases, chewing habit, other associated features, other adjunct treatment, prosthetic rehabilitation performed, technique used and follow-up.

## Results

### Literature search

**Fig 1:**
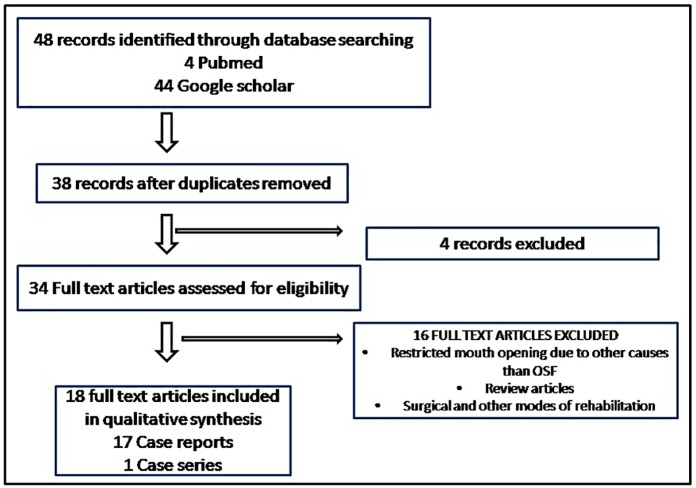
Flowchart of literature search.

The literature search revealed a total of 48 published articles in English (4 in PubMed and 44 in Google Scholar). The English language is generally perceived to be the universal language of science. However, the exclusive reliance on English-language studies may not represent all of the evidence. Excluding languages other than English may introduce a language bias and lead to erroneous conclusions. 10 duplicate articles were removed. Out of the 38 full-texts assessed for eligibility, 4 unrelated articles were excluded. Another 17 articles were excluded as they either were review articles, were not related to OSMF cases or did not mention prosthetic rehabilitation. Finally, 15 case reports and 2 case series reporting on a total of 21 unique cases were included [2,3,5–20]. (Fig 1). (S1 File)

A total of 21 cases were identified and analyzed from 17 papers published in the English-language literature. Of those, 9 cases employed the sectional denture technique, 4 cases emphasized the need-based treatment approach in which the conventional methods were modified, 4 cases used MEDs, and 1 case each used flexible dentures, oral screen prosthesis, oral stents, or surgery in conjunction with dentures. Selected characteristics of the included case reports and case series are shown in Table 1.

**Table 1:** Summary of data collected from case reports on prosthetic rehabilitation in oral submucous fibrosis patients.

S. No	Author	Year	No of patients	Chewing habits	Mouth opening	Other associated features	Surgery/ adjunct therapy	Treatment	Recall and follow-up
1	Kaira et al. [4]	2011	1	Chewing areca nuts with paan 4–5 times/day for 10 years	3.5 cm	Partially edentulous with angular cheilitis	No details	Sectional denture	No details
2	Sonune and Dange [5]	2012	1	Paan chewing 3–4 times a day for the last 15 years	2.5 cm	Completely edentulous	Saliva substitute	Need-based treatment approach: Use of small stock trays; medium-body elastomeric impression material was used for wash impression, and 2^nd^ molars were eliminated in tooth arrangement	Done. Denture adhesives were recommended.
3	Oswal et al. [6]	2013	1	Chewing betel nuts (along with tobacco and other unspecified ingredients) four to five times a day for 6 to 8 years	1.5 cm	Dentulous	Surgery done	Oral screen prosthesis (The oral screen appliance was intended to use as an oral stent along with mouth exercises to prevent relapse of the corrected mouth opening)	No details
4	Gajwani et al. [7]	2008	1	Betel nut chewing for 30 years	2.7 cm	Completely edentulous with salivary gland hypofunction	Physiotherapy, local topical corticosteroids, intralesional injection, multivitamin capsules, chlorhexidine mouth wash	Primary impression: single-stage peripheral tracing was accomplished with putty vinyl polysiloxane impression material. Secondary impression: light-body vinyl polysiloxane impression material. Semi-adjustable articulator was used to record jaw relation. Modification of conventional dentures.	No details
5	Saraf et al. [8]	2014	1	No details	2.2 cm	Partially edentulous	No details	Sectional denture	No details
6	Prasad et al. [9]	2012	1	25 years of areca nut/betel nut chewing	2 cm	Completely edentulous	Intralesional injections	Sectional denture	No details
7	Kumar et al. [10]	2014	1	Chewing areca nuts with paan 4–5 times/day for 7 years	3.1 cm	Completely edentulous	Intralesional corticosteroid and hyaluronidase Injections	Sectional impression tray technique	No details
8	Kajave et al. [11]	2015	1	Chewing tobacco for the past 25 years	2.8 cm	Completely edentulous	No details	Sectional custom tray, sectional denture base and fabrication of “customized hinge” for hinged mandibular denture.	Done for 6 months—satisfactory
9	Sheela et al. [12]	2015	1	Chewing tobacco for the past 20 years	2.8 cm	Completely edentulous	Surgery done	Silicon putty material—primary impression sectional custom tray, sectional denture base, and precision attachment applied to the sectional complete denture.	Evaluation was done at recall visits and adjustments were done as required
10	Krishna et al. [13]	2013	2	No details	Pt 1: 2 cm Pt 2: 2.4 cm	Partially edentulous	No details	Flexible impression tray techniques, catalyst proportion altered to reduce setting time, fabrication of two-piece custom sectional trays, use of locking button.	No details
11	Varghese et al. [14]	2014	1	Chewing tobacco with paan for the past 20 years	2 cm	Completely edentulous	Surgery	Sectional splint to maintain the mouth opening of the patient achieved after surgery.	Recalled for 6 months, vertical height maintained at 4 cm
12	Abbasali et al. [15]	2013	1	Chewing tobacco for the past 20 years	2.5 cm	Completely edentulous	Surgical grafts, mouth opening exercises, antibiotics	Surgery accompanied by the use of dentures to increase the vestibular depth.	No details
13	Caculo et al. [16]	2013	1	Chewing tobacco for the past 20 years	3 cm	Completely edentulous	No details	Sectional denture	No details
14	Sharma et al. [2]	2013	1	No details	2.5 cm	Completely edentulous	No details	Sectional and hinged complete dentures	Recalled up to 1 year, good results
15	Ibrahim et al. [17]	2012	1	Betel nut chewing for past 30 years	2.5 cm	Partially edentulous	Topical corticosteroids, intralesional steroid injection, antioxidants, immunomodulants, multivitamin capsules, iron supplements	Conventional dentures were modified by relining with permanent silicone soft liner material	No details
16	Vinayagavel et al. [18]	2014	1	No details	2.3 cm	Partially edentulous	No details	Sectional prosthesis with magnetic attachments	Recalled for 6 months—satisfactory results
17	Patil and Patil [19]	2012	4	No details	Pt 1: 2.8 cm Pt 2:2.9 cm Pt 3:3.4 cm Pt 4:3 cm	Dentulous	No details	Mouth-exercising device	Recalled for 6 months—satisfactory results

## Discussion

Oral submucous fibrosis (OSF) is a potentially malignant, chronic, progressive disorder seen mostly in people from Asia, where the habit of betel chewing is frequently practiced [20]. Most parts of the oral cavity are affected by fibrosis, which causes rigidity of the lips, tongue, palate, pharynx and even the upper third of the esophagus. The oral mucosa and the deeper tissues become stiffened due to subepithelial and submucosal myofibrosis, with increased limitations in mouth opening and tongue protrusion causing eating and swallowing difficulties along with phonation issues [21]. Different treatment approaches consisting of drug administration, surgical procedures and physiotherapy have been tried with varying success rates, but it still is quite challenging for a clinician to restore function and esthetics due to a patient’s clinical presentation [22].

### Epidemiological aspect

OSF has a high prevalence rate in South East Asian counties including India (0.62–6.42%), China (1–3.03% in the Hunan Province), Sri Lanka, Vietnam (0.15–14.4%) and Taiwan (0.086–17.6%) [23, 24]. Immigration of large number of Asian population to the western world has resulted in the increasing use of areca nut in these countries, especially among the youngsters. The increased prevalence is attributed to the growing popularity of areca nut based commercial products like pan masala. The increasing appeal of these products among youngsters is due to their easy availability, cost effectiveness and the form of marketing strategies employed to commercialize these products. Although OSF was considered as a predominantly Asian disease, the global immigration of Asians with areca nut associated habit and the increasing appeal of areca nut products among youngsters may eventually lead to the rise of OSF as a global disease [24, 25].

### Pathophysiology

The debilitating clinical features associated with OSF can be attributed to the sub epithelial fibrosis, which in turn is a result of an overall increased collagen production and decreased collagen degradation. Extracellular matrix is considered as a central core in the pathogenesis of OSF. OSF develops as a combined result of a number of individual biological pathways based on the effects of the different components of OSF [26]. The alkaloids in the areca nut results in an increased proliferation of fibroblast, which in turn results in an increased collagen production. The tannins and the catechins in the areca nut aid in the cross linking of collagen there by increasing the overall stability of collagen. As a result of these cross linking, the collagen is relatively more resistant to degradation, which in turn results in an increased accumulation of stabilized collagen [27]. Areca nut has a high copper content resulting in a rise in the levels of copper dependent enzyme lysyl oxidase. Lysyl oxidase, in turn, results in an overall increase in the collagen synthesis and cross-linking [28].

Areca nut is shown to stimulate pro-inflammatory cytokines and has a negative impact on the levels of anti-fibrotic molecules including interferon-γ [29]. Genetic polymorphisms of pro-fibrotic genes like tumor necrosis factor (TNF) β-1 can result in an overall increase in the proliferation of fibroblast [30]. Areca nut causes an overall decrease in collagen degradation due to reduced number of collagen phagocytic cells. The number of phagocytic cells is inversely proportional to the level of the arecoline. The areca nut users show a gross increase in the levels of the tissue inhibitors of matrix metalloproteinases (TIMP) and a decrease in the matrix metalloproteinases (MMPs). This in turn results in a continuous accumulation of ECM which eventually leads to the development of fibrosis [31].

### Diagnostic workup

A diagnosis of OSF can be rendered based on the clinical features and the history of associated areca nut use. The early symptoms include burning sensation, especially to spicy food, the formation of vesicles. As the lesion progresses, the mucosa loses its natural flexibility, becomes leathery and eventually develops clinically palpable fibrotic bands. Early histopathological features include sub-epithelial hyalinization, fibrosis, inflammation, and increased number of fibroblasts. The advanced histopathological features are characterized by degeneration and fibrosis of deeper structures with epithelial atrophy with an overall decrease in the cellular content [1, 32]. As a result of the fibrosis, there is a gross reduction in the mouth opening of OSF patients. Untreated OSF cases lead to progressive fibrosis with extensive involvement of oral cavity and esophagus. OSF cases may also show early signs of malignant transformation, characterized by the development of epithelial dysplasia and eventual development of frank squamous cell carcinoma [26].

### Surgical approaches and their apparent success/failure

Surgical relief of the fibrotic bands have shown significant immediate results. Unfortunately, the common post- surgical complication of surgically treated OSF cases, include the relapse of the fibrosis. The relapsing fibrosis may affect the oral cavity to a greater degree than the initial fibrosis. Non-surgical approach for OSF includes the use of fibrinolytic agents, use of mouth expansion devices, mouth opening exercise etc. These procedures have shown mixed results with most cases needing life- long treatment and follow up to prevent recurrences [32].

### Prosthetic rehabilitation

The patient is advised to discontinue the habit of betel chewing and advised to participate in physiotherapy (with maximum effort, the patient has to open and close the mouth wide for 15–20 minutes at least 3–4 times a day every day). The patient is instructed to use a saliva substitute before taking a primary impression for prosthetic rehabilitation. The ultimate prosthetic treatment is usually started after the required preliminary management [5].

A detailed recording of oral tissues is essential for the fabrication of oral prostheses. Modifications in conventional techniques and the use of flexible and sectional dentures, new materials, and magnetic attachments have been evaluated [3]. The various procedures and modifications used by dental practitioners to restore function and esthetics are described below.

## Types of trays

### Sectional stock tray for preliminary impressions

Prosthetic rehabilitation in an OSF patient is quite a challenge. In each case, the individual variables, such as the size and shape of the existing dental arch, position and state of the remaining teeth, and residual ridge resorption may cause difficulties in obtaining an acceptable preliminary impression with a conventional stock tray. It is challenging to make an accurate impression with a complete arch stock tray for OSF patients with restricted mouth opening [22].

Quality diagnostic casts and a well-fitted stock tray are critical for treatment planning in OSF patients with restricted mouth opening. Although there are individual anatomic discrepancies, this system allows different combinations of tray sizes and forms to be assembled into an anatomically well-fitted conforming tray. Modifications of conventional techniques include the usage of stock impression trays on each side of the mouth for sectional impressions with heavy- and light-body silicone impression materials as well as with a combination of the two. For primary impressions, flexible impression trays made using silicone putty or modeling plastic impression compound can be used for making sectional impressions. The two separate parts of the sectional custom tray are inter-connected with hinges, plastic building blocks, orthodontic expansion screws or locking levers connected only at the handle [3].

#### Challenges

Dental practitioners have encountered several problems with the use of sectional trays during the fabrication and placement of prostheses. When the strength of the connecting midline joint is inadequate, the impressions taken can become deformed. Polyether and polyvinyl siloxane are elastic impression materials that are self-adherent, and, when used for sectional placement, they require intraoral mechanical separation [3].

To overcome the challenges associated with sectional impression procedures, dissimilar materials such as irreversible hydrocolloid materials that are non-adherent can be used. The two separate halves of the elastic impression materials may be inserted and removed successfully. This system is currently under trial and is not yet commercially available [3].

#### Final impression

To minimize errors in the impressions that are caused by manipulation distortion after setting, a medium or light viscosity elastomeric impression material can be used.

### Flexible impression trays

To make an accurate diagnostic cast, a non-rigid, flexible tray should be used along with silicon putty impression material that can be molded within the mouth before it sets. The insertion and removal is easier in these cases as the silicon trays are flexible. Materials such as reversible and irreversible hydrocolloids, elastomeric impression materials, border molding materials such as modeling plastic, vinyl polysiloxane and polyethers can be used. The ease of manipulation of the material, accurate placement of the material onto the ridges/borders, and multiple insertion and removal of the border-molded impression tray can be reduced/eliminated. The definite accuracy and elimination of the use of water bath give elastomeric materials an advantage over impression compounds [3, 33].

### Mandibular swing-lock complete dentures

Collapsible mandibular swing-lock dentures with a cast cobalt-chromium framework, a lingual hinge and a conventional labial swing-lock can be used for prosthetic rehabilitation in OSF patients with restricted mouth opening. This helps these prostheses to be collapsible and maintain their structural durability. They are easy to insert and remove and provide maximum coverage for good support, retention and stability [3].

### Sectional complete denture with cast magnetic attachments

Sectional complete dentures consist of two left and right sections that are joined with an acrylic resin overlay to connect the four studs. These prostheses restore esthetics but cause a little discomfort due to restricted tongue space.

Excellent adhesion and attractive force can be achieved using Fe-Pt dental magnetic attachments and are clinically very useful. Commercially available prefabricated dental magnetic systems are available in several sizes. A castable magnetic attachment of any size or shape can be fabricated by casting it in a dental casting machine. These attachments are either placed on the root canal of the abutment tooth or implant abutment and rigidly connect the anterior segment to the posterior segment with its concavo-convex design. This prevents the wearing of magnetized components, averts denture deflection and decreases stress concentration at the lingual or palatal midline hinge during masticatory functions, thus minimizing breakage [3].

#### Graft stabilizing clip

In OSF patients undergoing surgery to correct mouth opening, Le and Gornitsky tried to use graft-stabilizing clips (GSCs) as oral stents for 6 months to prevent the relapse of the corrected mouth opening. This is a simple design that is easy to fabricate and ensures the positive contact of the graft with the recipient site. GSCs are a removable appliance that can be worn, removed and cleaned easily by the patient, making periodic examination of the surgical site possible [34, 35].

#### Postoperative oral physiotherapy aid for edentulous OSF patients

Achieving optimal mouth opening with the application of interocclusal forces form an integral part of the treatment of OSF patients. Many oral physiotherapy aids, from wooden spatulas to mouth gags (such as Heister or Ferguson), are currently in use. In edentulous OSF patients, there is transmission of the forces to the atrophic ridges, which can cause soft tissue injuries and fractures of these aids. As an adjunct to surgeries, custom-made occlusal splints with grooves are also advisable. Dentures with added occlusal rims help with maintaining normal mouth opening without requiring any patient compliance as they are to be worn only during physiotherapy sessions. These appliances are economical, comfortable and easy to maintain. After effective tissue healing, conventional dentures can be given [36–38].

#### Mouth-exercising device

Mouth-exercising devices (MED) have been introduced to increase elasticity by squeezing or stretching the buccal mucosa in OSF patients. These devices can be used alone or in combination with other treatment modalities, such as drug therapies and surgery. In mature scars, physiotherapy triggers the loosening of the thick fibrous tissue, causing the separation of collagen fibers from each other by pulsed ultrasound, which leads to increased pliability [18, 39–40].

## Conclusion

There is limited data about prosthodontic rehabilitation in edentulous and partially dentulous patients affected with OSF that help in terms of function and esthetics. A multifaceted therapy that includes the patient ceasing areca nut eating, using salivary substitutes, switching to a diet high in protein and minerals, taking vitamin B complex supplements, undergoing physiotherapy together with heat therapy such as hot rinses or lukewarm water, and/or participating in selective deep heating therapies such as shortwave and microwave diathermy should be employed together with prosthetic rehabilitation.

Careful treatment planning, cautious design and modifications of conventional dentures, and the use of sectional impression techniques can overcome the clinical difficulties associated with treating OSF cases. Periodic recalling, prosthesis maintenance and further design improvements help with keeping the prosthesis stable, functional and easy to use.

## Supporting information

S1 Table PRISMA checklist. (DOC)

S1 File First page of each cited papers. (DOCX)
