# Supplementary material for: Prosthetic rehabilitation of oral submucous fibrosis patients: A systematic review of published case reports and case series
Source: PLoS One. 2017 Sep 6;12(9):e0184041. doi: 10.1371/journal.pone.0184041 (PMC5587233; doi:10.1371/journal.pone.0184041)
Supplement: S1 File — (DOCX) [file pone.0184041.s002.docx]

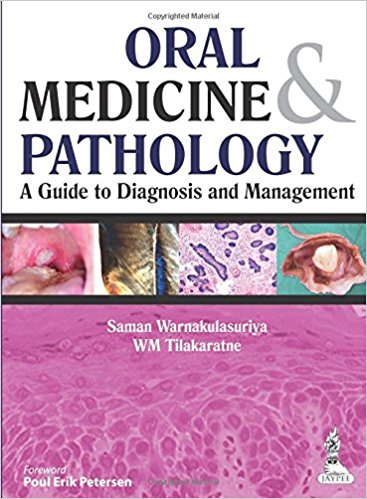


| \| **CASE REPORT** \| **[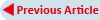](http://www.ijdr.in/article.asp?issn=0970-9290;year=2009;volume=20;issue=3;spage=365;epage=369;aulast=Pavesi)**[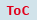](http://www.ijdr.in/showbackIssue.asp?issn=0970-9290;year=2009;volume=20;issue=3)**[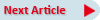](http://www.ijdr.in/article.asp?issn=0970-9290;year=2009;volume=20;issue=3;spage=374;epage=376;aulast=Pradeep)** \| \| --- \| --- \| \|  \| \| |
| --- | --- | --- | --- | --- |
| **Year** : 2009  \|  **Volume** : 20  \|  **Issue** : 3  \|  **Page** : 370-373 |
|  |
| Custom sectional impression trays with interlocking type handle for microstomia patients |
|  |
| [Aquaviva S Fernandes](http://www.ijdr.in/searchresult.asp?search=&author=Aquaviva+S+Fernandes&journal=Y&but_search=Search&entries=10&pg=1&s=0), [Kennedy Mascarenhas](http://www.ijdr.in/searchresult.asp?search=&author=Kennedy+Mascarenhas&journal=Y&but_search=Search&entries=10&pg=1&s=0), [Meena A Aras](http://www.ijdr.in/searchresult.asp?search=&author=Meena+A+Aras&journal=Y&but_search=Search&entries=10&pg=1&s=0) Department of Prosthodontics, Goa Dental College and Hospital, Goa, India  Click [**here**](http://www.ijdr.in/article.asp?issn=0970-9290;year=2009;volume=20;issue=3;spage=370;epage=373;aulast=Fernandes#cadd) for **correspondence address** and email   \| Date of Submission \| 17-Aug-2008 \| \| --- \| --- \| \| Date of Decision \| 29-Jan-2009 \| \| Date of Acceptance \| 25-Mar-2009 \| \| Date of Web Publication \| 30-Oct-2009 \| |
|  |
| **[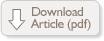](http://www.ijdr.in/article.asp?issn=0970-9290;year=2009;volume=20;issue=3;spage=370;epage=373;aulast=Fernandes;type=2)[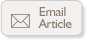](http://www.ijdr.in/emailArticle.asp?issn=0970-9290;year=2009;volume=20;issue=3;spage=370;epage=373;aulast=Fernandes)[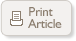](http://www.ijdr.in/printarticle.asp?issn=0970-9290;year=2009;volume=20;issue=3;spage=370;epage=373;aulast=Fernandes)[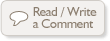](http://www.ijdr.in/readercomments.asp?issn=0970-9290;year=2009;volume=20;issue=3;spage=370;epage=373;aulast=Fernandes;aid=IndianJDentRes_2009_20_3_370_57371)[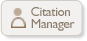](http://www.ijdr.in/citation.asp?issn=0970-9290;year=2009;volume=20;issue=3;spage=370;epage=373;aulast=Fernandes;aid=IndianJDentRes_2009_20_3_370_57371)** |
|  |
| \|   Abstract \|  \|  \| \| --- \| --- \| --- \|   Making impressions in microstomia patients is often cumbersome. A modification of standard impression procedure is often necessary while treating such patients. This article describes the fabrication of a custom sectional impression tray with interlocking type of a handle for definitive impression procedures in a microstomia patient.  **Keywords:** Microstomia, sectional trays, definitive impression procedures   \| **How to cite this article:** Fernandes AS, Mascarenhas K, Aras MA. Custom sectional impression trays with interlocking type handle for microstomia patients. Indian J Dent Res 2009;20:370-3 \| \| --- \|  \| **How to cite this URL:** Fernandes AS, Mascarenhas K, Aras MA. Custom sectional impression trays with interlocking type handle for microstomia patients. Indian J Dent Res [serial online] 2009 [cited 2017 Jul 31];20:370-3. Available from: <http://www.ijdr.in/text.asp?2009/20/3/370/57371> \| \| --- \| |
